# Latent Iron Deficiency as a Marker of Negative Symptoms in Patients with First-Episode Schizophrenia Spectrum Disorder

**DOI:** 10.3390/nu10111707

**Published:** 2018-11-08

**Authors:** Sung-Wan Kim, Robert Stewart, Woo-Young Park, Min Jhon, Ju-Yeon Lee, Seon-Young Kim, Jae-Min Kim, Paul Amminger, Young-Chul Chung, Jin-Sang Yoon

**Affiliations:** 1 Department of Psychiatry, Chonnam National University Medical School, Gwangju 61469, Korea; skyxmin@naver.com (M.J.); dabium@hanmail.net (J.-Y.L.); sykimpsy@chonnam.ac.kr (S.-Y.K.); jmkim@chonnam.ac.kr (J.-M.K.); 2 Mindlink, Gwangju Bukgu Community Mental Health Center, Gwangju 61220, Korea; fmjtt111@naver.com; 3 Institute of Psychiatry, Psychology and Neuroscience, King’s College London, London SE5 8AF, UK; robert.stewart@kcl.ac.uk; 4 South London and Maudsley NHS Foundation Trust, London SE5 8AF, UK; 5 Orygen, The National Centre of Excellence in Youth Mental Health, Centre for Youth Mental Health, The University of Melbourne, Parkville, VIC 3052, Australia; amminger@unimelb.edu.au; 6 Department of Psychiatry, Chonbuk National University Medical School, Jeonju 54907, Korea; chungyc@jbnu.ac.kr

**Keywords:** schizophrenia, psychosis, iron, ferritin, negative symptom, first-episode

## Abstract

Iron deficiency may alter dopaminergic transmission in the brain. This study investigated whether iron metabolism is associated with negative symptoms in patients with first-episode psychosis. The study enrolled 121 patients with first-episode schizophrenia spectrum disorder, whose duration of treatment was 2 months or less. Negative symptoms were measured using the Positive and Negative Syndrome Scale (PANSS) and Clinician-Rated Dimensions of Psychosis Symptom Severity (Dimensional) scale of the Diagnostic and Statistical Manual of Mental Disorders, Fifth Edition (DSM-5). Prominent negative symptoms were defined as moderate or severe negative symptoms on the Dimensional scale of the DSM-5. Iron deficiency was defined as a serum ferritin ≤ 20 ng/mL. Patients with iron deficiency were significantly more likely to have prominent negative symptoms (45.2 vs. 22.2%; *p* = 0.014) and a higher PANSS negative symptoms score (*p* = 0.046) than those with normal ferritin levels. Patients with prominent negative symptoms had significantly lower ferritin levels (*p* = 0.025). The significance of these results remained after controlling for the duration of illness and other confounding variables. Our finding of an independent association between iron deficiency and negative symptoms in patients at the very early stage of illness implies that iron dysregulation has an effect on negative symptoms in patients with schizophrenia. The possibility of therapeutic intervention with iron should be further investigated.

## 1. Introduction

Schizophrenia is a severe mental illness that typically begins in adolescence or early adult life and is often chronic and disabling. Although delusions and hallucinations are the typical symptoms seen at the time of diagnosis, negative symptoms such as avolition or diminished emotional expression are more persistent core symptoms. The revised dopamine hypothesis, i.e., prefrontal hypodopaminergia, is one of the most influential theories regarding the etiology of schizophrenia [1]. Generally, the prefrontal dopamine system suppresses the limbic dopamine system; however, in patients with schizophrenia, this suppression seems to be reduced due to disrupted prefrontal dopaminergic activity, leading to elevated limbic dopaminergic activity. Additionally, prefrontal hypodopaminergia is concordant with negative symptoms of schizophrenia [2].

Iron is an essential trace element for nearly all living organisms and is a component of hemoglobin, which is vital for the delivery and storage of oxygen [3]. Iron is also required for cell viability, as it is a constituent of proteins involved in DNA synthesis, cell proliferation, and energy metabolism [4]. Furthermore, iron is the most abundant transition metal in the brain, and is vital for a number of neurological functions including neurotransmitter synthesis, myelination of neurons, mitochondrial function, and electron transfer [5,6,7]. Therefore, a sufficient iron supply is necessary for neurodevelopmental processes [8]; in fact, reductions in the iron supply at several stages of development result in long-term changes in monoamine neurotransmission that outlast the iron deficient periods [9,10,11]. Conversely, iron overload can cause cellular toxicity and neuronal damage via free radical formation and peroxidation of lipid membranes [3,12]. Iron accumulates as the brain ages and may be linked to motor and cognitive dysfunction in the elderly [6].

Iron homeostasis is essential for the integrity of the brain monoaminergic system [13], and its dysregulation has been reported to be involved in neuropsychiatric disorders associated with dopamine, such as attention deficit hyperactivity disorder and Tourette syndrome [14,15,16,17], and neurological movement disorders such as restless legs syndrome (RLS) and Parkinson’s disease, which are associated with reduced central dopamine activity [18]. In patients with RLS, the levels of iron and ferritin in serum or cerebrospinal fluid are significantly reduced [18,19,20]. Ferritin levels were also reported to be positively correlated with central nervous system (CNS) dopamine levels in Parkinson’s disease [21].

In previous animal studies, induced iron deficiency has been reported to alter both dopaminergic and serotoninergic transmission in the brain [22]. This iron-dopamine interaction might therefore conceivably account for symptoms in patients with schizophrenia. Some evidence suggests a role for iron deficiency in chronic and tardive akathisia, which is associated with reduced dopamine activity due to the use of dopamine antagonists. Results from previous studies investigating the association between akathisia and iron deficiency in patients with psychosis have been inconsistent; in patients with chronic psychotic disorders, akathisia was found to be associated with decreased plasma ferritin and iron levels [23,24,25,26,27], whereas no association between iron indices and akathisia was observed in other studies of chronic and acute akathisia [28,29,30,31].

In some studies, decreased serum iron levels have been observed in catatonic and acutely psychotic presentations [32,33]. However, compared with other psychiatric and neurological disorders related to dopamine neurotransmission, there is a lack of evidence for a role of iron metabolism in the psychopathology of patients with first-episode schizophrenia. Recently, it was shown that iron depletion in monkeys disrupted both dopaminergic and serotoninergic transmission in various CNS regions, including the striatum and prefrontal cortex [13]. Therefore, negative symptoms in schizophrenia might potentially be attributed at least in part to brain iron homeostasis breakdown, which has a widespread effect on the monoaminergic system. However, to our knowledge, no investigation has focused on the association between iron metabolism and negative symptoms associated with prefrontal hypodopaminergic state in patients with first-episode schizophrenia.

Iron depletion occurs in the brain before it occurs in red blood cells during progressive negative iron balance [34]. Therefore, a latent iron deficiency state may cause depletion of brain tissue without depleting red blood cells [35]. In this study, we hypothesized that iron dysregulation may be associated with negative symptoms of schizophrenia spectrum disorder. Specifically, we investigated the associations of serum iron and related variables, including ferritin levels, with negative symptoms in patients with first-episode psychosis. Ferritin is of particular utility as a primary marker of iron metabolism because it regulates the binding and storage of iron and plays an important role in maintaining iron metabolism homeostasis and regulating iron content in the brain.

## 2. Methods

### 2.1. Study Population

We analyzed data from an early psychosis cohort assembled at Gwangju, Republic of Korea (Gwangju Early Treatment and Intervention Team; GETIT study). The GETIT cohort included patients whose duration of treatment for psychotic symptoms was ≤2 years and who met the criteria for ‘schizophrenia spectrum disorder and other psychotic disorders’ according to the Diagnostic and Statistical Manual of Mental Disorders, Fifth Edition (DSM-5) [36]. Inclusion criteria for this study were a first episode of schizophrenia, schizophreniform disorder, or other specified schizophrenia spectrum disorder and duration of treatment ≤ 2 months to minimize the confounding effects of illness on iron metabolism. Exclusion criteria were age less than 18 years and diagnosis of a substance- or medication-induced psychotic disorder, psychotic disorder due to another medical condition, or severe neurological or medical disorders. This study was conducted from September 2015 to August 2018 and was approved by the Chonnam National University Hospital Institutional Review Board. All subjects provided written informed consent before participation.

### 2.2. Sociodemographic and Clinical Data

The baseline data included age, sex, diagnosis, type of antipsychotics taken, duration of treatment, and duration of untreated psychosis (DUP), which was defined as the time between the appearance of the first psychotic symptoms and the start of appropriate antipsychotic treatment [37].

Psychopathology including negative symptoms was measured by the Positive and Negative Syndrome Scale (PANSS) [38,39]. We used the five factors of the PANSS; positive, negative, depressive, cognitive, and excited factors [40]. Additionally, prominent negative symptoms were defined as moderate or severe on the negative symptom dimension of the Clinician-Rated Dimensions of Psychosis Symptom Severity (Dimensional) scale in the DSM-5 [36]; moderate or severe decrease in facial expressivity, prosody, gestures, or self-initiated behavior. Calgary Depression Scale for Schizophrenia (CDSS) [41,42], and Social Occupational Functioning Assessment Scale (SOFAS) [43]. All raters were trained psychiatrists and were certified to rate the PANSS. The dietary habits of patients were measured using a 20 item self-administered questionnaire based on dietary guidance published by the Korean Ministry of Health and Welfare [44]. The dietary questionnaire consists of three subcategories, including five items for diet regularity (e.g., regular diet, breakfast every morning, appropriate amounts), six items for a balanced diet (e.g., dairy foods, fruits, vegetables), and nine items for an unhealthy diet and habits (e.g., instant food, fatty foods, salty foods, caffeine-containing foods). Each item was scored on three Likert scales (1, 3, and 5 points) according to the frequency of the dietary habit. Higher scores indicate better dietary habits in each category.

### 2.3. Laboratory Measures

Fasting venous blood was drawn in the morning of the assessment day. The iron parameters measured comprised serum ferritin, iron, total iron binding capacity (TIBC), transferrin saturation (iron/TIBC), and hemoglobin levels. We used ferritin, as a key measure of iron status, to predict negative symptoms. In this study, latent iron deficiency was defined as a serum ferritin ≤ 20 ng/mL [45,46].

### 2.4. Statistical Analyses

Demographic and clinical characteristics were compared according to the presence of iron deficiency defined by a serum ferritin ≤ 20 ng/mL and prominent negative symptoms, using the chi-square test, independent t-test, and Mann–Whitney U-test, as appropriate. The difference in PANSS scores for negative factors between the two groups according to iron deficiency was compared using analysis of covariance (ANCOVA) after adjusting for other significant variables (i.e., those with statistical significance in the univariate analyses and sex). Finally, logistic regression analysis was used to investigate associations between prominent negative symptoms and iron deficiency after adjusting for significant variables. Values that were not normally distributed were entered as covariates after log transformation. All statistical tests were two tailed, and a *p*-value < 0.05 was considered to indicate statistical significance. All analyses were performed with IBM SPSS Statistics software version 23.0. 

## 3. Results

The GETIT cohort enrolled 257 patients with psychosis for 3 years beginning in 2015. Of these, 194 patients (75.5%) were in their first episode. After excluding subjects based on criteria regarding duration of treatment, age, and diagnosis, this study recruited 121 patients whose first-episode psychosis had been treated for 2 months or less. Seventy-three patients (60.3%) were female. The median (interquartile range, IQR) age of the participants was 27.0 (22.0–32.0) years. The median (IQR) duration of treatment and DUP were 1.0 (0.7–1.2) and 2.2 (1.0–12.0) months, respectively. Prominent negative symptoms were observed in 31 (25.6%) of patients, with no significant sex difference. Serum ferritin ≤ 20 ng/mL were observed in 34 (28.1%) patients and were very significantly more common in female patients (Table 1). Median serum ferritin and mean hemoglobin levels were significantly lower in female patients than in male patients [21.1 (12.7–47.8) ng/mL vs. 111.2 (77.2–165.5) ng/mL and 12.5 ± 1.4 g/dL vs. 14.7 ± 1.1 g/dL, respectively; both *p*-values < 0.001]. Transferrin saturation was also significantly lower in female patients than in male patients [24.1 ± 14.2 vs. 35.5 ± 15.1%; *p*-value < 0.001].

Table 1 compares the sociodemographic and clinical characteristics of the participants according to the presence of iron deficiency and prominent negative symptoms. Patients with ferritin level ≤ 20 ng/mL with had significantly lower hemoglobin, iron, and transferrin saturation levels, and higher TIBC. Patients with low ferritin levels were significantly more likely to have prominent negative symptoms (45.2 vs. 22.2%; *p*-value = 0.014) and higher PANSS negative symptoms scores score (*p*-value = 0.046) than those with normal ferritin levels. Patients with iron deficiency had a significantly shorter duration of treatment. Patients with prominent negative symptoms had significantly higher CDSS scores and PANSS negative, positive, depressive, cognitive, and total scores. They also had significantly lower SOFAS scores. Patients with prominent negative symptoms had significantly lower ferritin levels. The DUP was significantly longer and they were significantly less likely to receive inpatient service at the time of assessment.

**Figure 1:**
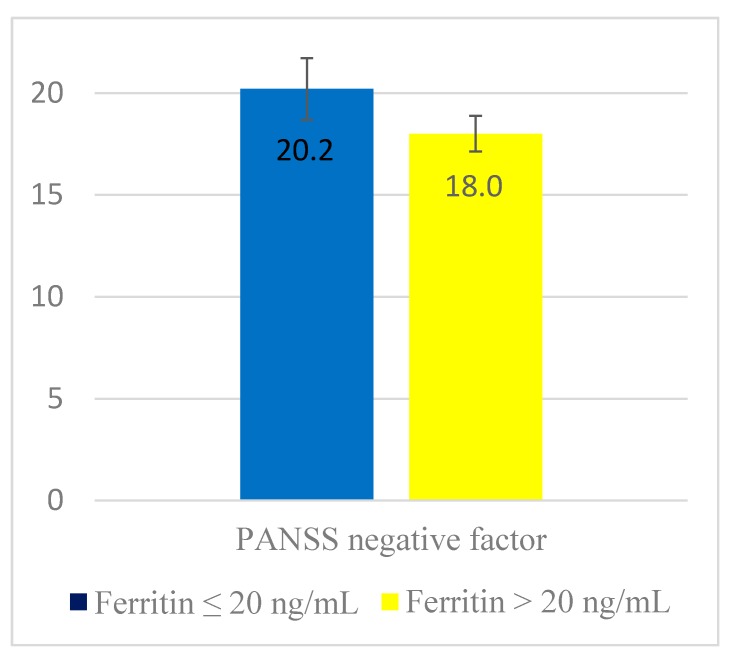
Adjusted mean (95% confidence interval) of Positive and Negative Syndrome Scale (PANSS) negative factor score according to iron deficiency. Adjustment for sex, inpatient status, duration of untreated psychosis, duration of treatment, and scores on the Social Occupational Functioning Assessment Scale, Calgary Depression Scale for Schizophrenia, and PANSS positive and cognitive factors. F = 5.471, *p*-value = 0.021.

Figure 1 shows the results of the ANCOVA for differences in the PANSS negative symptom score according to the presence of iron deficiency after controlling for the confounding effects of sex, inpatient status, DUP, duration of treatment, and scores on the SOFAS, CDSS, and PANSS positive and cognitive factors. After controlling for these confounding factors, the PANSS negative symptoms score was significantly higher in patients with a ferritin level ≤ 20 ng/mL (*p*-value = 0.021). Logistic regression analyses also showed that prominent negative symptoms were independently associated with the low ferritin group [OR (95% CI) = 9.2 (1.8–47.0); *p*-value = 0.008] and log-transformed ferritin level [OR (95% CI) = 5.7 (1.1–29.8); *p*-value = 0.041], even after controlling for the above confounding variables.

## 4. Discussion

Among the patients with first-episode psychosis, those with latent iron deficiency defined by a low serum ferritin level had significantly more severe negative symptoms. The association between iron status and negative symptoms was still significant after controlling for the duration of illness and other confounding variables. These results support our hypothesis that abnormal iron metabolism decreases dopaminergic activity and is consequently associated with negative symptoms. To the best of our knowledge, this is the first study to investigate an association between iron dysregulation and negative symptoms in first-episode schizophrenia spectrum disorder.

The association of interest between iron status and negative symptoms was derived from an analysis of cross-sectional data; therefore, the direction of causation cannot be concluded with certainty. Specifically, it is possible that iron depletion is a cause of negative symptoms, as will be considered further, or that negative symptoms cause iron depletion. In patients with chronic psychotic disorders, iron deficiency may be attributed to behaviors such as poor nutritional intake, in turn due to psychotic disorders, or to environmental factors such as long-term hospitalization. However, the effects of chronic psychotic experiences on iron status might not be critical because all participants in this analysis were experiencing the first episode of psychosis and their illness was at a very early stage. In addition, eating patterns and behaviors did not differ significantly according to the presence of iron deficiency and prominent negative symptoms. Therefore, the effect of mental disorders on general diet quality might not be significant. The fact that statistical significance remained after controlling for DUP, a potential factor mediating the association between iron status and negative symptoms, also suggests that iron metabolism may predict negative symptoms, and may not be a consequence of psychotic symptoms in patients with schizophrenia.

Negative symptoms have been found to be associated with reduced dopamine activity [2] and it is possible that iron deficiency might act to increase the likelihood of these symptoms through a range of possible mechanisms. First, iron is a co-factor of tyrosine hydroxylase, which acts as the rate-limiting enzyme in dopamine synthesis [47]. Therefore, reduced brain iron levels would be expected to reduce the availability of iron in dopamine neurons, which may in turn reduce dopamine activity in the CNS.

Second, iron depletion has been found to mimic dopamine type 2 (D2) receptor blockade in numerous animal models [23]. It has been hypothesized that iron deficiency may alter D2 receptor function, because iron is part of the D2 receptor. Additionally, it has been suggested that, as the D2 receptor is an iron-containing protein, low serum iron levels result in hypofunctionality of D2 receptors, which predisposes patients with schizophrenia to akathisia and negative symptoms [27,48,49]. Some studies have reported that nutritional iron deficiency alters dopaminergic neurotransmission, increasing the concentration of extracellular dopamine and reducing the activity of dopamine transporters and D2 receptors in the striatum [50], an essential integrative node in the dopamine transmission pathway, and striatal dopaminergic dysfunction has been linked to cortical dopaminergic dysregulation [51]. Iron deficiency may therefore be linked to cortical dopamine dysregulation, which may cause negative symptoms in schizophrenia.

Third, hypoferremia, a cytokine-mediated reduction of circulating non-heme iron in the blood, is a common response to systemic infections or generalized inflammatory disorders. Inflammation-induced hypoferremia may disrupt brain development, leading to functional defects in adulthood that are synonymous with psychiatric disorders such as schizophrenia 8 [52]. Emerging literature provides evidence that gestational exposure to infection contributes to the etiology of schizophrenia [53]. During prenatal inflammation, hypoferremia plays a fundamental role in the developmental effects of this maternal insult to mesolimbic dopamine function [8].

Several previous studies of the general population have observed a relationship between low iron status and mood, indicating a potential role for iron in the development of mild depressive symptoms [54,55,56]. In other studies of the geriatric population or stroke patients, high iron levels were associated with depression. In this study, however, we observed no relationship between iron status and depression in patients with schizophrenia.

Negative symptoms are a significant barrier to successful functional outcomes and recovery in individuals with schizophrenia [57]. However, currently available treatments for negative symptoms appear to confer only modest benefits [58]. A study reported akathisia in a patient with iron deficiency that had not responded to standard interventions, but responded dramatically to intravenous iron treatment [59]. Therefore, longitudinal research is needed to clarify the relationship between negative symptoms of schizophrenia and iron status, and the possibility of therapeutic intervention should also be investigated.

Several limitations should be considered when interpreting our results. First, the association between serum iron and dopamine metabolism in the brain was not supported by the study data. In addition, serum iron measurements may not accurately indicate brain iron stores. There is little direct evidence to suggest that circulating iron levels reflect brain iron concentrations, although a previous magnetic resonance imaging (MRI) study showed that the proton transverse relaxation rate in brain regions is correlated with systemic iron status [60]. Therefore, our hypothesis and explanation of the data should be carefully interpreted. Further studies should consider direct measurement methods with high sensitivity and specificity to accurately assess brain iron levels [61]. Second, serum ferritin is a marker of inflammation as well as iron stores [62]. Therefore, serum ferritin levels can vary depending on various conditions, including inflammatory status. In addition, transferrin saturation calculated as the iron/TIBC ratio may not be a perfect marker of iron deficiency due to the contribution from a certain portion of non-transferrin bound iron [63]. Therefore, confounding factors must be considered in measuring circulating iron deficiency. Various markers that more precisely reflect iron status should be used in future studies. Third, iron intake was not directly measured in this study. Finally, although all of the study participants were experiencing their first episode of psychosis, the duration of psychotic illness and treatment may show an association with iron deficiency. However, the types of antipsychotics used did not differ significantly according to iron status, and the statistical significance of this result was consistent after controlling for DUP and duration of treatment. Further longitudinal research is needed to determine the causal relationship between iron status and psychopathology.

## 5. Conclusions

We found that patients with prominent negative symptoms had significantly lower ferritin levels. Multivariate analyses adjusting for the duration of illness, depression, psychotic symptoms, social function, and other confounding variables showed that latent iron deficiency was significantly associated with negative symptoms, implying a potential relationship between iron dysfunction and negative symptoms in patients with schizophrenia spectrum disorder. The possibility of therapeutic intervention with iron should be further investigated.

## Figures and Tables

**Table 1:** Comparisons of sociodemographic and clinical characteristics according to the presence of iron deficiency and prominent negative symptoms.

		Serum Ferritin Level				Prominent Negative Symptoms			
	Total (*N* = 121)	≤20 ng/mL (*N* = 34, 28.1%)	>20 ng/mL (*N* = 87, 71.9%)	Statistical Value	*p*-Value	Yes (*N* = 31, 25.6%)	No (*N* = 90, 74.4%)	Statistical Value	*p*-Value
Sociodemographic and clinical characteristics									
Age, Med, (IQR) year	27.0 (22.0–32.0)	27.5 (22.8–32.5)	25.0 (21.0–32.0)	U = −1.270	0.204	25.0 (20.0–30.0)	27.0 (22.0–32.5)	U = −1.403	0.161
Sex, female; *N* (%)	73 (60.3)	33 (45.2)	40 (54.8)	χ^2^ = 26.653	<0.001	21 (28.8)	52 (72.2)	χ^2^ = 0.957	0.328
DUP, Med. (IQR) Mo.	2.2 (1.0–12.0)	4.0 (1.0–24.0)	2.0 (1.0–11.0)	U = −0.937	0.349	8.5 (1.1–25.0)	2.0 (1.0–7.3)	U = −2.859	0.004
Duration of Tx, Med. (IQR) Mo.	1.0 (0.7–1.2)	1.0 (0.5–1.0)	1.0 (0.8–1.5)	U = −2.512	0.012	1.0 (0.8–1.2)	1.0 (0.6–1.2)	U = −0.102	0.919
Inpatient status, *N* (%)	71 (58.7)	17 (23.9)	54 (76.1)	χ^2^ = 1.469	0.226	12 (16.9)	59 (83.1)	χ^2^ = 6.854	0.009
Diagnosis, *N* (%)				χ^2^ = 0.240	0.887			χ^2^ = 5.613	0.060
Schizophrenia	77 (63.6)	21 (27.3)	56 (72.7)			24 (31.2)	53 (68.8)		
Schizophreniform	32 (26.4)	10 (31.3)	22 (68.6)			7 (21.9)	25 (78.1)		
Other specified.	12 (9.9)	3 (25.0)	9 (75.0)			0 (0.0)	12 (100.0)		
Antipsychotics, *N* (%)				χ^2^ = 6.843	0.233			χ^2^ = 7.713	0.173
Amisulpride	32 (26.4)	8 (25.0)	24 (75.0)			12 (37.5)	20 (62.5)		
Aripiprazole	21 (17.4)	10 (47.6)	11 (52.4)			2 (9.5)	19 (90.5)		
Paliperidone	53 (43.8)	14 (26.4)	39 (73.6)			15 (28.3)	38 (71.7)		
Risperidone	5 (4.1)	0 (0.0)	5 (100.0)			0 (0.0)	5 (100.0)		
Quetiapine	7 (5.8)	1 (14.3)	6 (85.7)			1 (14.3)	6 (85.7)		
None	3 (2.5)	1 (33.3)	2 (66.7)			1 (33.3)	2 (66.7)		
Laboratory measures									
Hemoglobin, mean (SD) g/dL	13.4 (1.7)	12.5 (10.5- 13.2)	13.9 (13.1–14.9)	t = −6.966	<0.001	12.9 (2.2)	13.6 (1.4)	t = −1.703	0.097
Iron, Med. (IQR) μg/dL	90 (58–115)	51 (26–90)	100 (67–127)	U = −4.671	<0.001	73 (40–112)	91 (61–116)	U = −1.416	0.157
TIBC, Med. (IQR) μg/dL	326 (291–354)	360 (333–396)	310 (280–340)	U = −4.994	<0.001	338 (285–367)	325 (292–350)	U = −1.146	0.252
Transferrin Sat., mean (SD) %	28.6 (15.6)	16.8 (11.0)	33.2 (14.7)	t = −5.908	<0.001	25.8 (15.9)	29.5 (15.4)	t = −1.138	0.257
Ferritin, Med. (IQR) ng/mL	48.7 (18.0–105.5)	12.4 (7.6–16.0)	86.6 (43.7–128.5)	U = −8.529	<0.001	29.3 (8.8–89.2)	64.6 (20.9–109.4)	U = −2.238	0.025
Ferritin ≤ 20 ng/mL, N (%)	34 (28.1)	Not applicable				14 (41.2)	20 (58.8)	χ^2^ = 6.005	0.014
Psychiatric measures									
PANSS, Positive, mean (SD)	16.0 (4.8)	16.7 (4.6)	15.7 (4.9)	t = 1.031	0.305	19.2 (3.9)	14.8 (4.7)	t = 4.682	<0.001
Negative, mean (SD)	18.6 (5.6)	20.3 (5.8)	18.0 (5.4)	t = 2.021	0.046	23.7 (5.6)	16.9 (4.5)	t = 6.809	<0.001
Cognitive, mean (SD)	14.4 (3.7)	14.3 (3.9)	14.5 (3.6)	t = −0.222	0.825	15.8 (3.1)	13.9 (3.8)	t = 2.439	0.016
Depressive, mean (SD)	12.4 (3.7)	12.3 (3.3)	12.5 (3.8)	t = −0.222	0.825	14.7 (3.3)	11.6 (3.5)	t = 6.326	<0.001
Excited, mean (SD)	7.5 (3.0)	7.8 (3.1)	7.3 (3.0)	t = 0.778	0.438	8.4 (3.6)	7.1 (2.7)	t = 1.766	0.085
Total, mean (SD)	68.8 (15.1)	71.2 (15.0)	67.8 (15.2)	t = 1.112	0.268	81.6 (12.5)	64.3 (13.4)	t = −5.833	<0.001
Prominent negative Sx, *N* (%)	31 (25.6)	14 (45.2)	17 (54.8)	χ^2^ = 6.005	0.014	Not applicable			
SOFAS, mean (SD)	58.2 (10.2)	56.9 (9.7)	58.7 (10.4)	t = −0.872	0.385	50.4 (10.8)	60.9 (8.5)	t = −5.550	<0.001
CDSS, mean (SD)	4.5 (4.0)	4.2 (3.8)	4.6 (4.1)	t = −0.484	0.629	7.3 (4.7)	3.6 (3.3)	t = 4.063	<0.001
Diet habit,									
Regular diet, mean (SD)	17.0 (6.5)	17.1 (6.7)	17.0 (6.5)	t = 0.126	0.900	16.1 (6.4)	17.3 (6.6)	t = −0.904	0.368
Balanced diet, mean (SD)	18.2 (6.8)	18.7 (7.4)	18.0 (6.6)	t = 0.490	0.625	18.2 (6.8)	18.2 (6.9)	t = 0.016	0.987
Healthy diet, mean (SD)	31.8 (6.3)	32.0 (5.6)	31.8 (6.6)	t = 0.168	0.867	31.6 (6.0)	31.9 (6.4)	t = −0.241	0.810

Med. Median; IQR, Interquartile Range; SD, Standard Deviation; Mo., month; DUP, Duration of Untreated Psychosis; Other specified., other specified schizophrenia spectrum disorder; PANSS, Positive and Negative Syndrome Scale; SOFAS, Social Occupational Functioning Assessment Scale; CDSS, Calgary Depression Scale for Schizophrenia; Tx, treatment; Sx, symptom; Sat., saturation.
